# 25-Hydroxycholesterol in health and diseases

**DOI:** 10.1016/j.jlr.2023.100486

**Published:** 2023-12-16

**Authors:** Cindy Nguyen, Julien Saint-Pol, Shiraz Dib, Caroline Pot, Fabien Gosselet

**Affiliations:** 1 UR 2465, Laboratoire de la Barrière Hémato-Encéphalique (LBHE), Univ. Artois, Lens, France; 2 Department of Clinical Neurosciences, Laboratories of Neuroimmunology, Service of Neurology and Neuroscience Research Center, Lausanne University Hospital and University of Lausanne, Lausanne, Switzerland

**Keywords:** 25-hydroxycholesterol, oxysterols, 7α,25-dihydroxycholesterol, cholesterol, reverse cholesterol transfer, ABCA1, LXR

## Abstract

Cholesterol is an essential structural component of all membranes of mammalian cells where it plays a fundamental role not only in cellular architecture, but also, for example, in signaling pathway transduction, endocytosis process, receptor functioning and recycling, or cytoskeleton remodeling. Consequently, intracellular cholesterol concentrations are tightly regulated by complex processes, including cholesterol synthesis, uptake from circulating lipoproteins, lipid transfer to these lipoproteins, esterification, and metabolization into oxysterols that are intermediates for bile acids. Oxysterols have been considered for long time as sterol waste products, but a large body of evidence has clearly demonstrated that they play key roles in central nervous system functioning, immune cell response, cell death, or migration and are involved in age-related diseases, cancers, autoimmunity, or neurological disorders. Among all the existing oxysterols, this review summarizes basic as well as recent knowledge on 25-hydroxycholesterol which is mainly produced during inflammatory or infectious situations and that in turn contributes to immune response, central nervous system disorders, atherosclerosis, macular degeneration, or cancer development. Effects of its metabolite 7α,25-dihydroxycholesterol are also presented and discussed.

Cholesterol is the dominant sterol in animal cells where it can be found in all membranes. For example, 3–6% of the cellular cholesterol is detected in the endoplasmic reticulum (ER) membrane, and almost 60–90% in the plasma membrane (PM) (1). Cholesterol ensures proper membrane integrity, fluidity, and biochemical functions, such as protein activity and signal transduction processes. All these processes affect endocytosis, cell growth, death, and proliferation. The membrane cholesterol pool is also important in host defense processes during immune responses with an increasing role highlighted by studies, indicating that macrophages and other immune cells adapt their cholesterol metabolism to ensure their effector functions (reviewed in (2)). In the central nervous system (CNS), cholesterol is enriched in myelin sheath and essential for synaptogenesis and neuron functioning (3). Beside to these essential roles, cholesterol is also precursor to bile acids, to vitamin D, and to a variety of steroid hormones.

Even though cholesterol concentrations in cells and in the whole animal remain essentially constant, it has been demonstrated that 7–9% of the whole-body sterol pool has a daily turnover in small animals like the mice and almost 0.7% in larger animals with a lower metabolic rate like humans (3). In fact, total intracellular pool of cholesterol is a balance between *i*) synthesis, *ii*) production/uptake of lipoproteins by a process named reverse cholesterol transport (RCT), *iii*) storage under esterified forms in intracellular lipid droplets, or *iv*) metabolization via oxidation processes (Fig. 1). Cholesterol synthesis and RCT will be described later in detail in this review. Esterification by the enzyme acetyl-CoA acetyltransferase (ACAT) in ER occurs when intracellular cholesterol levels reach a threshold level (4). The cholesterol is an unsaturated lipid, thus is very sensitive to oxidation. Its metabolization results in oxysterol formation that is early intermediates in the metabolism of cholesterol to bile acids. These latter represent an elimination form of cholesterol, as well as a solubilization and transport system for cholesterol in the biliary tract and for lipids in the small intestine (5).

**Fig. 1:**
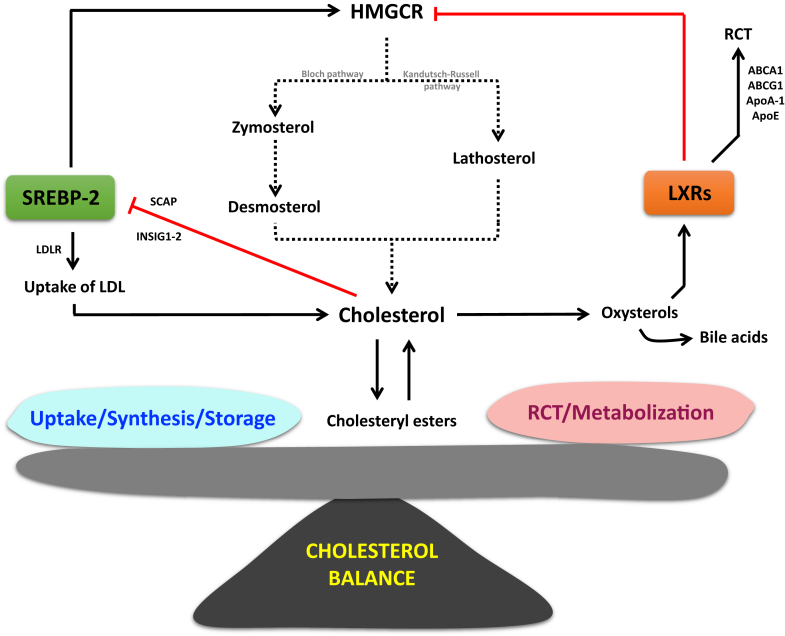
Regulation of intracellular cholesterol pools. Circulating low-density lipoproteins (LDL) are recognized and internalized by LDL receptor (LDLR) expressed at the cell plasma membrane (PM). It starts the endocytosis process that leads to transfer the cholesterol to late endosomes and subsequently to the lysosomes. Cholesterol is then transferred to the PM where it participates to membrane fluidity, signaling processes, etc. Excess of PM cholesterol is transferred to high density lipoproteins (HDL) by the transporter ABCA1 through the reverse cholesterol transfer (RCT) process. ABCA1 can also flip cholesterol from the inner PM sheath to the outer sheath. Excess of cholesterol might be also metabolized into oxysterols with many of them that are ligands for the liver X Receptor (LXR) pathway. Activation of LXR by oxysterols increase ABCA1 expression and then cholesterol release to HDL. If the PM cholesterol content remains elevated, cholesterol is transferred to the endoplasmic reticulum (ER) by Aster proteins. This triggers the trapping of the SREBP cleavage–activating protein (SCAP)/sterol response element–binding protein 2 (SREBP-2) complex within the ER that it abolishes the transcriptional expression of LDLR and 3-hydroxy-3-methylglutaryl-coenzyme A reductase (HMGCR), the rate-limiting enzyme responsible for cholesterol synthesis. ABCA1, ATP-binding cassette subfamily A member 1.

The oxysterol family comprises almost 60 members of 27-carbon oxidized derivatives of cholesterol and is produced by a variety of cells of our body via auto-oxidation, enzymatic activity or via these both processes simultaneously (6). In addition, some oxysterols are also present in diet or can be generated during digestion, heating, or prolonged storage of cholesterol-containing products (7). Considered for long times just as “cholesterol metabolites,” it is now well accepted that some of these oxysterols play key roles in cholesterol metabolism, where they can regulate cholesterol synthesis and RCT. However, they are increasingly associated with a wide variety of other cell functions like cell death, cell proliferation and differentiation, and immune response. Therefore, this is not surprising that the important role for several oxysterols have been recently highlighted in subsets of cancers, as well as in different diseases such as neurodegenerative or neuroinflammatory diseases, cancers, and atherosclerosis.

Among the different identified oxysterols, 25-hydroxycholesterol (25-HC) received attention for its importance in intracellular cholesterol metabolism, as well as in cell migration, in physiological barrier tightness, or in immune response. This review aims thus to bring the latest knowledge about 25-HC synthesis and roles in different physiological processes related to human health and diseases.

## Cholesterol synthesis

As mentioned above, intracellular pool of cholesterol depends on the closely regulated balance between cholesterol synthesis, uptake, storage, RCT, and metabolization (Fig. 1).

Briefly, cholesterol is synthesized from acetyl-CoA in two major synthesis pathways (Bloch and Kandutsch-Russell pathways), involving at least 20 enzymes (reviewed in (8, 9)). The rate-limiting enzyme of these biosynthesis pathways is located in the ER and is the 3-hydroxy-3-methylglutaryl-coenzyme A reductase (HMGCR; EC 1.1.1.34) that catalyzes the conversion of 3-hydroxy-3-methylglutaryl-CoA into mevalonic acid. Importantly, this enzyme, as all the enzymes involved in the cholesterol synthesis, are regulated by the sterol response element binding protein 2 (SREBP-2) pathway (10, 11).

SREBP-2 is organized into three segments. An NH_2_-terminal domain of almost 485 amino acids that contains the basic-helix-loop-helix-leucine-zipper (bHLH-Zip) motif able to bind DNA, two hydrophobic transmembrane-spanning segments interrupted by a short loop of about 31 amino acids that project into the lumen of the ER, and a COOH-terminal regulatory domain of almost 585 amino acids. The NH_2_- and COOH-terminal segments project into the cytoplasm.

SREBP-2 is produced in the ER and its C-terminal domain immediately binds to the transport protein SREBP cleavage-activating protein (SCAP) (11). When intracellular cholesterol ER concentrations are low (<5% mole of ER cholesterol), the SREBP-2/SCAP complex translocates to the Golgi where SREBP-2 undergoes two cleavage reactions catalyzed sequentially by the serine protease S1P (site 1 protease) and the metalloprotease S2P (site 2 protease) to release an active transcription factor corresponding to the NH_2_ domain, which penetrates to the nucleus and activates target gene expression (11, 12, 13, 14, 15). The N- and C-terminal domains of SREBP are then degraded by proteasomal degradation, whereas SCAP cycles back to the ER where it takes part in new rounds of escorting SREBP-2.

*HMGCR* expression is controlled by SREBP-2 pathway, which also regulates the expression of the low density lipoprotein receptor (*LDLR*) (11). Uptake of low density lipoprotein (LDL) by LDLR represents a mechanism by which cells can also increase their cholesterol content (1, 16). Briefly, when LDL particles bind the LDLR, they are internalized by endosomes and are degraded within lysosomes. Then the cholesterol is transferred to the PM, thus increasing lipid concentration (17). When cholesterol concentrations in PM increases, in particular in the inner leaflet, Aster proteins and mainly Aster B protein transfer cholesterol from PM to ER through PM-ER membrane contact sites (18). Note that oxysterols such as 25-HC can compete with this cholesterol transfer process (18). In the ER, cholesterol concentrations increase and when are > to 5% mole of ER lipids, binds to insulin-induced genes 1 and 2 (INSIG1 and INSIG2) which trap the SREBP-2/SCAP complex in this organelle, thus impeding its translocation to the Golgi and subsequently its cleavage. It abolishes the *HMGCR* and *LDLR* transcription, and thus leads to a decrease of the cholesterol synthesis and uptake (11, 19).

## LXR signaling pathway and the RCT

In parallel to the SREBP-2 pathway, another major signaling pathway sensing the intracellular pool of cholesterol is the liver X receptor (LXR) pathway. Two LXR isoforms have been identified: LXRα encoded by the *NR1H3* gene and LXRβ encoded by the *NR1H2* gene. Despite their high sequence homology (77%), the expression of these isoforms is variable in different organs (20, 21). Indeed, LXRα is highly detected in the liver, intestine, kidney adipose tissue, and lungs. In CNS, LXRβ is ubiquitously expressed while LXRα is preferentially expressed in microglial cells and neurons of the subcortical zone (22, 23, 24, 25).

From N terminus to the C terminus, these receptors are divided into six domains from A to F. The A/B-domains comprise the first regulatory domain (AF-1) and are sensitive to coactivators. The C domain is the DNA-binding domain LXR interacting with response elements that consists of a direct repeat of the core sequence 5′-AGGTCA-3′ spaced by four nucleotides (DR4) (26). The D domain is a hinge region interacting with corepressors in the absence of LXR ligand but also involved in dimerization because LXR form a permissive dimer with the retinoid X receptor α. Interestingly, LXRα is able to act as monomer to regulate the renin and c-myc genes expression by interacting with a *cis*-acting DNA element known as the CNRE (an overlapping cAMP response element and a negative response element) region (27). The second regulatory domain (AF-2) is the domain E that interacts with the ligands via a ligand-binding pocket structure. The F-domain only exists in some nuclear receptor subfamily and its functions remain unknown in LXRs.

Subsets of oxysterols were subsequently identified few years later as natural ligands for these nuclear receptors (28) and several in vitro and in vivo studies have demonstrated that oxysterols like 24S-HC, 24(S),25-epoxycholesterol, 25-HC, or 22(R)-HC activate LXR functions with different affinities (28, 29). Cholesterol precursors, zymosterol and desmosterol, also are ligands for LXRs (30). Noteworthy, 25-HC showed highest ability to interact and activate LXRα than LXRβ in transfected CV-1 cells (fibroblasts isolated from *Cercopithecus* aethiops monkey kidneys) (28).

In the presence of their ligands or inflammatory stimuli like tumor necrosis factor α, LXR directly trigger the expression of specific target genes regulating cholesterol homeostasis (29, 31, 32). Among the target genes controlled by LXR, the ATP-binding cassette subfamily A member 1 (*ABCA1*) transporter is the most studied and consequently the best characterized (33). ABCA1 is a lipid transporter present at the PM and transferring lipids, essentially cholesterol but also phospholipids, to lipid-poor or lipid-free APOA-I particles thus generating pre-β high density lipoproteins (HDL) (34). In the CNS, these particles are mainly composed of APOE (35). These poorly lipidated particles are subsequently lipidated by ABCG1 and to some extend by ABCG5 and ABCG8 to form mature HDL that are then transported into the bloodstream and are taken in charge by the liver (35, 36). This process is then termed RCT for reverse cholesterol transfer. ABCA1 also transfers cholesterol from the inner leaflet of the PM to the outer leaflet in order to maintain a low inner leaflet cholesterol level (37). The inducible degrader of the LDL-receptor is an E3 ubiquitin ligase downregulating LDLR levels. *Inducible degrader of the* *LDL-receptor* and *SREBP-1c* are also target genes of the LXR signaling pathway (38, 39). Therefore, by controlling the cholesterol content in PM, the LXR/ABCA1 axis is considered as a key regulator of the intracellular cholesterol levels. *L*xr*α/β* KO animals display several organs defects and show deep CNS and behavioral abnormalities (40, 41, 42). In humans, deficiencies in ABCA1 functions and expression provoke Tangier disease that is characterized by low plasmatic concentrations of HDL, thus provoking cholesterol accumulation within tissues and cardiovascular defects like atherosclerotic lesions (43). In animal models, mimicking the neurodegenerative disorder Alzheimer’s disease (AD), stimulating LXR/ABCA1 axis with agonists (T0901317 or GW3965), or increasing Abca1 expression alleviates the disorder, while deleting Abca1 expression aggravates the β-amyloid burden which is characteristic of AD (44, 45, 46). Interestingly, the GWAS has recently identified *ABCA1* as a risk factor for AD, highlighting once again the importance of the LXR/ABCA1 axis in AD (47).

Despite the correlations between human disease and the LXR/ABCA1 axis, the mechanism of action of this latter remains unclear. In addition to its role in brain cholesterol homeostasis, LXR/ABCA1 axis is suspected to play a role in anti-inflammatory responses as well as immune reactions. *Abca1* KO mice display neuroinflammation and astrogliosis (48), while *Abca1* overexpression in reactive astrocytes improves the phagocytosis process (49). As an agonist of the LXR pathway, the role of the oxysterol 25HC in inflammatory processes and immune responses is discussed in details in the following paragraphs.

## Oxysterol synthesis: focus on 25-HC production

As developed above, excess of cholesterol in cells is oxidized to form oxysterols. Oxysterols directly produced from cholesterol are classiﬁed into side chain and ring-modiﬁed oxysterols. These oxysterols can be further modified to form secondary oxysterols that display more than one modiﬁcation, including modiﬁcations of hydroxyl, epoxy, keto, or hydroperoxy groups.

25-HC (cholest5-en-3β,25-diol) was one of the first commercially available oxysterol. Human tissues contain very low levels of 25-HC, much lower than, for example, 27-HC or 24S-HC. 25-HC is complexed and transported by lipoproteins (50) and its plasmatic concentration is usually only few ng/ml (51).

**Fig. 2:**
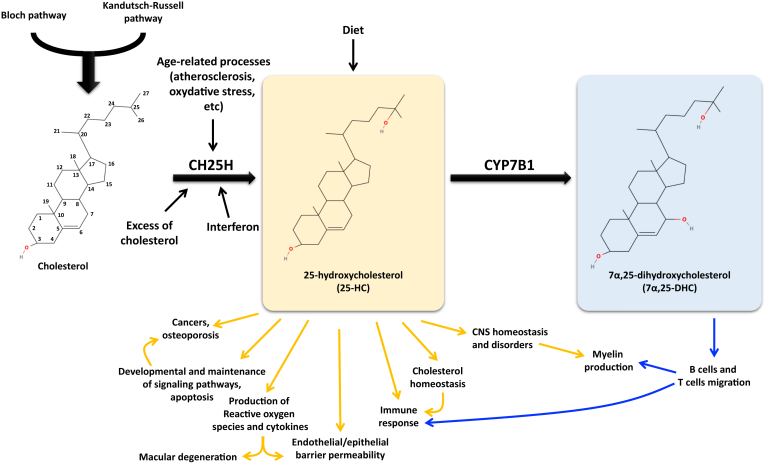
Production and roles of 25-hydroxycholesterol and 7α,25-dihydroxycholesterol. 25-hydroxylase (CH25H) converts cholesterol into 25-hydroxycholesterol (25-HC), which is then converted into 7α,25-dihydroxycholesterol (7α,25-DHC) by cytochrome P450 family 7 subfamily B member 1 (CYP7B1). 25-HC can also be provided by the diet. Many studies report effects of 25-HC and 7α,25-DHC in several cellular processes thus underlying their involvement in atherosclerosis, in cancers onset and development, immune response, and in neurodegenerative disorders.

**Fig. 3:**
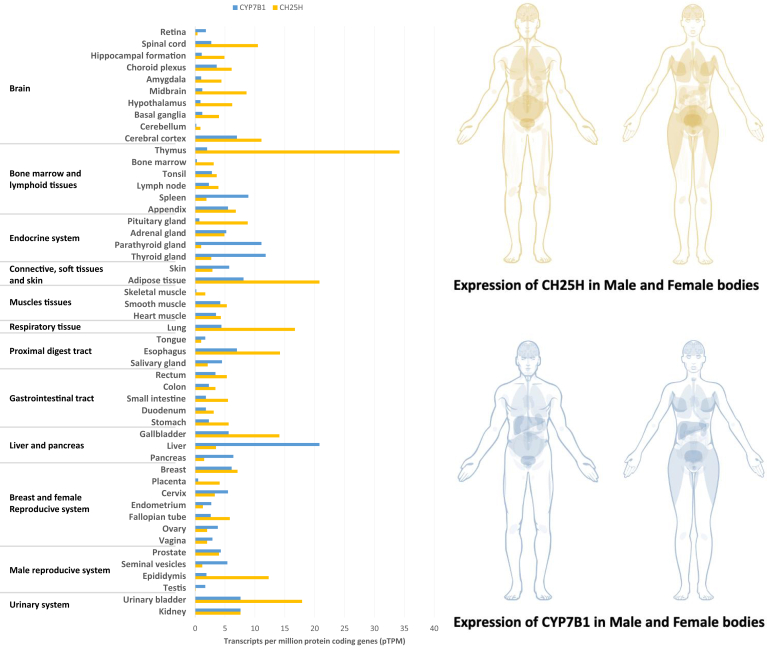
Expression patterns of *CH25H* and *CYP7B1* transcripts in different organs and in males and females. These data were obtained from Human Protein Atlas Website consulted in September 26th 2023. Consensus transcript expression levels summarized per gene in 50 tissues based on transcriptomics data Human Protein Atlas RNA-seq and The Genotype-Tissue Expression project. The consensus normalized expression (“nTPM”) value is calculated as the maximum nTPM value for each gene in the two data sources. For tissues with multiple subtissues (brain regions, lymphoid tissues, and intestine) the maximum of all subtissues is used for the tissue type. The tab-separated file includes Ensembl gene identifier (“Gene”), analyzed sample (“Tissue”) and normalized expression (“nTPM”). The data is based on The Human Protein Atlas version 23.0 and Ensembl version 109 (56).

25-HC can be generated by both enzymatic and nonenzymatic pathways (52) (Fig. 2). Enzyme responsible for 25-HC biogenesis is mainly the 25-hydroxylase (CH25H), which uses cholesterol and molecular oxygen as substrates and NADPH as a cofactor. This enzyme is expressed at low levels in several cell types, including hematopoietic cells, epithelial, and endothelial cells (ECs), but also in macrophages and lymphoid organs (53, 54, 55) (Fig. 3). Human atlas also reports high expression of CH25H in adipose tissues, lung, urinary bladder, and in gallbladder (Fig. 3). In CNS, where cholesterol metabolism is of prime importance for brain functioning, *Ch25h* is expressed by microglial cells (57, 58), astrocytes (59) and ECs of the blood-brain barrier (BBB) (58, 60). Expression pattern of CH25H can slightly vary between males and females as illustrated in Fig. 3.

Interestingly, CH25H expression and 25-HC production are closely related to the inflammatory and immune situations of the organs or tissues. *Ch25h* gene has been identified as an interferon-stimulated gene (61). Its expression is quickly increased in the heart, brain, muscle, kidney, lung, lymphoid organs, and most notably, liver upon in vivo exposure to a toll-like receptor 4 (TLR4) agonist, thus leading to a 25-HC increase in tissues and blood (62). Similarly, injection of a TLR4 ligand [like lipopolysaccharide (LPS), for example] into human subjects produces a transient increase in serum 25-HC (63). In addition, in vitro, production and secretion of 25-HC are observed in several cell lines including mouse macrophages, ECs, dendritic cells, astrocytes, and microglial cells treated with inflammatory molecules (64, 65, 66). Interestingly, it has been demonstrated that CH25H and LXR expressions are controlled by krüppel-like factor 4, a central anti-inflammatory transcription factor, in ECs and macrophages in vitro and in vivo models of atherosclerosis, thus suggesting an atheroprotective role of the krüppel-like factor 4–Ch25h/LXR axis (67).

Besides, other enzymes belonging to the cytochrome P450s such as CYP27A1, CYP46A1, and CYP3A4 can also produce small amounts of 25-HC (68, 69, 70). 25-HC can be further metabolized into 7α,25-dihydroxycholesterol (7α,25-DHC) by the enzyme sterol 7α-hydroxylase (CYP7B1), which also participates in bile acid synthesis (Fig. 3). This is important to note that expression patterns of CYP7B1 and CH25H do not fit very well as observed in many brain regions (Fig. 3). On the contrary to *CH25H*, *CYP7B1* is highly expressed in liver and in thyroid.

25-HC can also be 3-sulfated by sulfotransferases such as SULT2B1b to form 25HC3S that shows altered biological effects in comparison with 25-HC (71).

25-HC is one of the most detected oxysterols in case of long storage of cholesterol-rich products but is poorly produced in lipoproteins incubated in oxidative conditions in comparison with other nonenzymatically produced oxysterols like the 7-ketocholesterol (72). This indicates that cholesterol autoxidation is of limited importance for the formation of 25-HC in living organisms, but that 25-HC coming from dietary intake might be taken into account in studies using animals or patients.

## Role of 25-HC in cholesterol metabolism

As mentioned above, several oxysterols have been shown to be able to modulate LXR functions like 24S-HC, 24(S),25-epoxycholesterol, 25-HC, or 22(R)-HC and are thus considered as powerful regulators of cholesterol homeostasis (28, 29). However, data reported for 25-HC was first difficult to interpret and its direct participation in the regulation of cholesterol homeostasis was regularly debated. First, in mice, the basal expression of *Ch25h* is barely detected in the liver, a tissue with active cholesterol and fatty acid synthesis, but is detected in the lung, heart, and kidney. Secondly, disruption in the cholesterol *Ch25h* gene does not modulate cholesterol metabolism in mice (73). Then, patients with hereditary spastic paresis (SPG5), a rare inherited disease with mutations in the *CYP7B1* gene have highly elevated levels of 25-HC and 27-HC but normal levels of cholesterol and bile acids (74). All together these data challenged the hypothesis that 25-HC is of great importance for maintaining cholesterol homeostasis in vivo.

It is noteworthy that from another side, expression of *Ch25h* and 25-HC production both increase quickly in mouse adipose tissue and in rat liver when animals are fed with a high-fat diet (75, 76). In human visceral adipose tissue samples, 25-HC levels correlate with the body mass index (76) and inhibitory effects of 25-HC on adipogenic differentiation in C3H10T1/2 cells have been reported (77). But the major argument in favor of a direct role of 25-HC in cell cholesterol homeostasis is that injection of this oxysterol in animals suppress liver HMGCR expression and therefore cholesterol synthesis (75). The effect of 25-HC on HMGCR expression is now very well documented and strongly suggests that this oxysterol is still an important component in the control of the intracellular cholesterol metabolism, in interplay with inflammation status. In fact, when intracellular cholesterol pool is high, a constant outflow of cholesterol ensures an increase of this lipid in ER (18). Because CH25H is also expressed in ER, it leads to the conversion of cholesterol into 25-HC, even when the enzyme is expressed at low levels. 25-HC subsequently stimulates the binding of INSIG1 and INSIG2 proteins to HMGCR, leading to its ubiquitination and its subsequent transport from ER to cytosol where its proteosomal degradation occurs (78). 25-HC also promotes association of INSIG1 and INSIG2 to the SCAP/SREBP-2 complex and prevents its translocation to the Golgi, thus impeding the regulation of SREBP-2–dependent *HMGCR* and *LDLR* expression (79). In few words, a high intracellular concentration of cholesterol increases 25-HC production which in turn decreases HMGCR levels by two different molecular mechanisms, and therefore cholesterol synthesis. By blocking the SCAP/SREBP-2 pathway, 25-HC also blocks the LDL uptake. Therefore, increasing 25-HC synthesis leads to a decrease of cholesterol synthesis and uptake.

As mentioned previously, CH25H levels are largely influenced by inflammatory stimuli. A large body of evidence suggests now that 25-HC and its metabolite 7α,25-DHC both play a key role in the cholesterol metabolism reprogramming in responses to immune and inflammatory situations. Therefore, by regulating intracellular and membrane levels of cholesterol as mentioned above, 25-HC protects cells from infection rather than directly regulating lipid homeostasis in alive animals. These roles of 25-HC in immune functions are described in the following parts.

## 25-HC and the host defense

Despite that immune activities have also been observed for other oxysterols such 7-ketocholesterol or 27-HC, 25-HC and its metabolite 7α,25-DHC are now widely considered as the main oxysterols involved in immune functions (80).

Interferon signaling pathway constitutes the ﬁrst line of defense against many invading pathogens like viruses. As mentioned above, *CH25H* is an interferon-stimulated gene (61). In fact, 25-HC inhibits a variety of viruses’ infection by modulating sterol synthesis and distribution in cells. Several in vitro studies have shown that 25-HC inhibits infection by multiple enveloped viruses, including Kaposi’s sarcoma herpesvirus, human immunodeﬁciency virus type 1, Ebola virus, West Nile Virus, Zika virus, etc (reviewed in (81)). In return, some of these viruses can probably downregulate 25-HC production to facilitate their infection process as demonstrated in ECs infected by Kaposi’s sarcoma herpesvirus (82).

Interestingly, role of 25-HC in coronavirus disease 2019 infection has been very well documented and this oxysterol plays a key role in protection against this virus, suggesting that it can be a therapeutic target in future drugs development against severe acute respiratory syndrome coronavirus 2 (SARS-CoV-2). Thus, CH25H expression is found to be upregulated in macrophages and lung epithelial cells (EPs) gathered in bronchioalveolar lavage fluid from coronavirus disease 2019 patients (83), and 25-HC level is elevated in some patients suffering from SARS-CoV-2 infection (84). Recently, it has been demonstrated that 25-HC inhibits RNA-dependent RNA polymerase and main protease of the SARS-CoV-2 virus that are both key players of viral transcription and replication (85). However, this inhibitory mechanism deserves further investigations.

Action of 25-HC in infection by nonenveloped viruses remains more conflicting with studies reporting positive antiviral action, while others did not observe any effect (86).

**Fig. 4:**
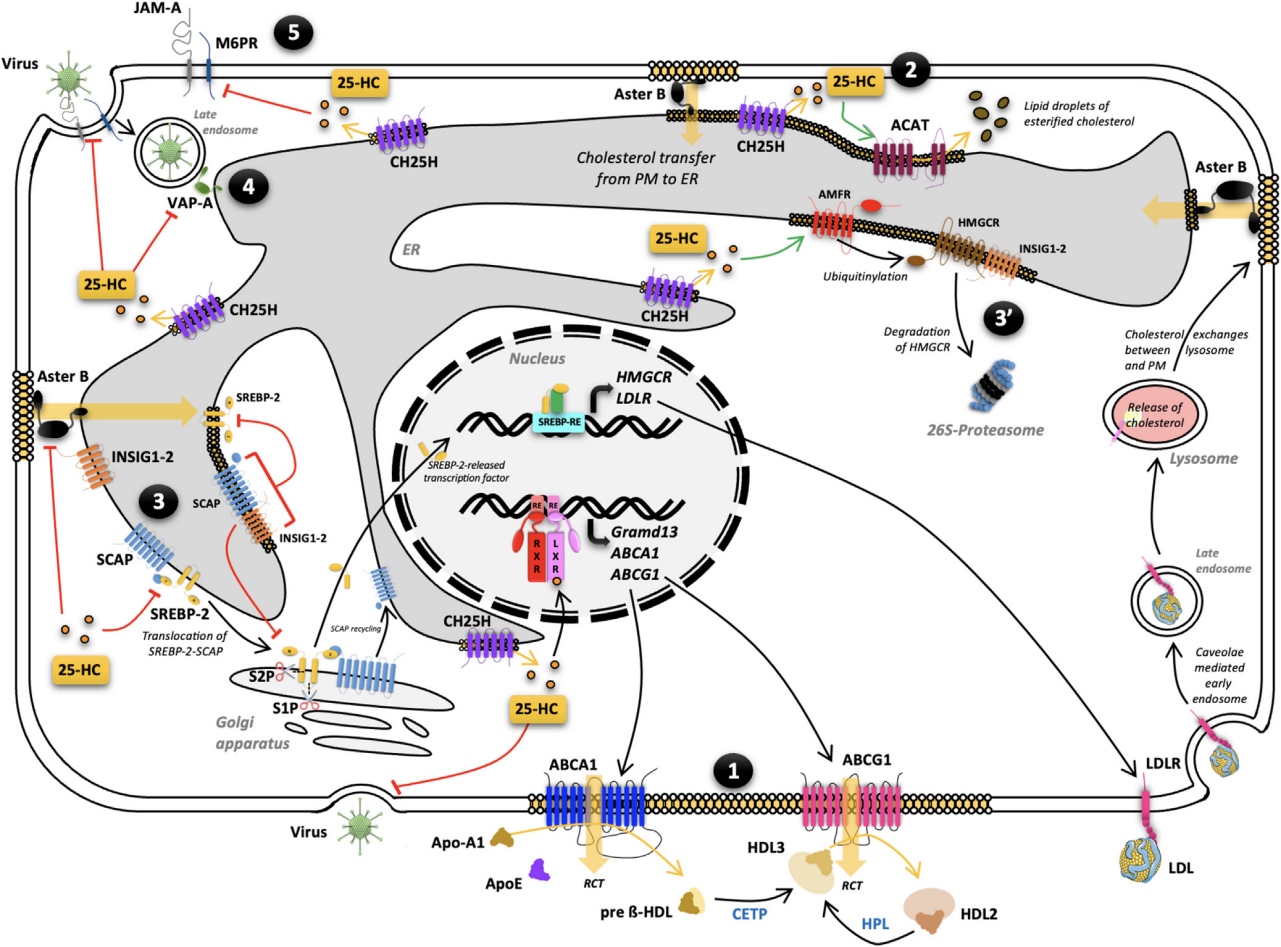
Molecular mechanisms of 25-HC protection against virus infection. 25-HC plays a key role by restricting virus fusion with the plasma membrane (PM) and entrance within cells. Several molecular mechanisms have been reported. The first molecular mechanism decreases PM cholesterol composition and consists to activate the LXR signaling pathway, and then the ABCA1-mediated cholesterol efflux (1). 25-HC also depletes this accessible cholesterol of the PM by activating the ACAT. This enzyme is located into the endoplasmic reticulum (ER) and esterifies cholesterol. Esterified cholesterol is then stored in cytosolic lipid droplets that decrease ER cholesterol that is then replaced by cholesterol transferred from PM (2). The third mechanism consists of sequestration of SREBP-2 into the ER via interacting with INSIG1/2 and SCAP, thus blocking SREBP-2 cleavage and the subsequent transcription of *HMGCR* and *LDLR* (3). 25-HC also promotes HMGCR interaction with INSIG1/2 that it leads to its ubiquitinylation by the E3 ubiquitin ligase autocrine motility factor receptor (AMFR) and proteosomal degradation (3′). Then, 25-HC modulates activity of cellular mediators like oxysterol-binding protein (OSBP) and the vesicle-associated membrane protein-associated protein-A (VAP-A), that provokes disturbances in the recycling of cholesterol between the ER and the late endosomes with the consequence to block the viral particles inside these vesicles, and do not allow their replications in the cytosol (4). Lastly, it has been recently reported that 25-HC down-regulates the junction adhesion molecule-A (JAM-A) and the cation independent isoform of mannose-6-phosphate receptor (M6PR), two crucial molecules essential for infection of a variety of viruses (5). 25-HC, 25-hydroxycholesterol; ABCA1, ATP-binding cassette transporter subfamily A member 1; ACAT, acetyl-CoA acetyltransferase; HMGCR, 3-hydroxy-3-methylglutaryl-coenzyme A reductase; INSIG, insulin-induced gene; LDLR, low density lipoprotein receptor; LXR, liver X receptor; SREBP-2, sterol response element binding protein 2.

Knowing the importance of the cholesterol levels for different steps of the viral infection, it has been demonstrated that 25-HC blocks viruses at different steps in their viral cycle infection (illustrated in Fig. 4).

1 First, 25-HC modifies PM cholesterol composition (87). By activating the LXR signaling pathway, and then ABCA1-mediated cholesterol efflux, 25-HC activates the RCT, thus resulting in a decrease of the cholesterol level at the PM, impeding the viral membrane fusion with cells.
2 Abrams *et al.* then suggested that 25-HC also depletes this accessible cholesterol via ACAT, thus leading to cholesterol esterification (88, 89). Esterified cholesterol is then stored in cytosolic lipid droplets. By activating ACAT, 25-HC provokes a decrease of cholesterol in ER which is then replaced by cholesterol transferred from the ER membrane. As the RCT, it leads to a decrease of the cholesterol content of the PM and thus decreases the ability of the virus to fuse with the PM, as demonstrated with human coronaviruses in lung adenocarcinoma cells (83).
3 Then, as described above, 25-HC sequesters SREBP-2 into the ER via interacting with INSIG1/2 and SCAP, thus blocking SREBP-2 cleavage and the subsequent transcription of *HMGCR* and *LDLR*. Remarkably, 25-HC also promotes HMGCR interaction with INSIG1/2 that it leads to its ubiquitinylation by the E3 ubiquitin ligase autocrine motility factor receptor (78). HMGCR is then expelled from the ER and is degraded by the proteasome (90, 91). Therefore, uptake of cholesterol is abolished, and cholesterol synthesis is decreased.
4 Furthermore, 25-HC modulates the activity of cellular mediators like oxysterol-binding protein and the vesicle-associated membrane protein-associated protein-A (VAP-A), which provokes disturbances in the recycling of cholesterol between the ER and the late endosomes with the consequence to block the viral particles inside these vesicles and do not allow their replications in the cytosol (92).
5 Lastly, it has been recently observed in ECs that 25-HC downregulates the junction adhesion molecule-A and the cation-independent isoform of mannose-6-phosphate receptor, two crucial molecules essential for infection of a variety of viruses (93). In addition, 25-HC actives the RIG-I signaling and downstream genes thus triggering an antiviral response (94).

Cholesterol present in PM is also targeted by bacteria that use this sterol, as the viruses, to infect the mammalian cells. Cholesterol-dependent cytolysins are pore-forming toxins secreted by bacteria that need membrane cholesterol for their effector function. By maintaining a low pool of accessible cholesterol in the PM, macrophages and neutrophiles are protected against cholesterol-dependent cytolysins (2, 14, 87, 95, 96). It is therefore likely that 25-HC will also protect cells against pathogen producing these toxins via mechanisms highlighted above.

Besides the direct effects of 25-HC on viral/bacterial infectious process, it has been showed that *Ch25h* mice show an increase in fatty acid desaturase 2 expression (58), which promotes the production of anti-inflammatory lipids (97), that can be an additional role of 25-HC in host defense mechanism. It has also been reported that 25-HC, through LXR pathway, downregulates interleukin (IL)-10 secretion in murine IL-27–induced Treg (98) and from human Th1 cells (30), thus highlighting a proinﬂammatory role of 25-HC in ﬁne-tuning CD4+ T-cell polarization (99).

Another process by which 25-HC regulates immune response is via its metabolite, the 7α,25-DHC produced by CYP7B1. 7α,25-DHC is a natural ligand for the previously orphan G-protein–coupled receptor Epstein Barr–induced G-protein–coupled receptor 2 (EBI2, also known as GPR183). This receptor is known to be important for adaptative immunity. In vitro, 7α,25-DHC binds to EBI2 (100) and stimulates the migration of EBI2-expressing mouse B cells and T-cells with half-maximum effective concentration values around 500 pM but had no effect on EBI2-deficient cells (101, 102). In vivo, 7α,25-DHC also promotes migration of LPS-activated B cells, CD41 and CD81 T-cells, and dendritic cells. Blocking of 7α,25-DHC synthesis by using the CYP7B1 inhibitor clotrimazole produces similar results than the use of EBI2-deficient cells (101). The source of 7α,25-DHC is stromal cell subsets present the lymphoid tissues (53). Protein expression of EBI2 is higher on human memory CD4^+^ T-cells than naïve CD4^+^ T-cells and 7α,25-DHC preferentially drives activated IL-17A–producing CD4^+^ T-cell trafficking (103).

## 25-HC as the first line of defense in the lungs

Besides the role of 25-HC and 7α,25-DHC in immune response, several studies have reported a key role of these oxysterols in lung in response to respiratory infection. In mouse, high expression of *Ch25h* is observed in resident alveolar macrophages (54). When respiratory infection is mimicked by injection of LPS, *Ch25h* expression is induced particularly in pulmonary ECs and alveolar macrophages (54, 104) and 25-HC levels are increased in lungs and bronchioalveolar lavage fluids (105). High-dose LPS increases 25-HC levels and provokes a loss of the lung endothelial barrier integrity by disorganizing VE-cadherin assembly (54). Interestingly, *Ch25h* KO mice show less damage than WT mice after high-dose LPS, but also less cytokines release suggesting that they are protected during lung injury. However, these latter observations seem to be LXR-independent (54). Alternatively, low dose of LPS activates LXR signaling pathway that induces apoptotic cells clearance by alveolar macrophages (104).

Lastly, it was also reported that 7α,25-DHC decreased the LPS-induced neutrophil increase in the alveolar space but did not show significant effects on the other inflammatory parameters studied (105).

Altogether, these data highlight dual roles for 25-HC in lung tissue homeostasis during low- versus high-injury states. This suggests that it might be challenging to target this pathway in lung disease and that further studies are needed particularly in human to better understand if 25-HC production needs to be increased or decreased to alleviate respiratory disease progression.

## Effects of 25-HC on vascular EC and EP

As detailed previously, 25-HC plays a key role in viral/bacteria defense and these studies have been performed in macrophages but also in ECs that line blood vessels. Macrophages and ECs represent the first line of defense against pathogen infection and tightly communicate together. EPs of the intestine are also suggested as the first line of defense in case of oral infection. Thus, several studies have focused on effects of 25-HC on ECs and EPs and their relationship with macrophages.

25-HC produced by macrophages accumulates in human coronary atherosclerotic lesions and promotes vascular inflammation and remodeling (106, 107). Studies aiming to better deciphering these 25-HC/EC interactions used human umbilical vein ECs or pulmonary ECs and confirmed that high concentrations of 25-HC have deleterious effect on these cells and affects endothelial barrier permeability. Thus, treatment of human umbilical vein ECs with 25 μM of 25-HC decreases the cell index (108, 109) and promotes production of a long list of inflammatory molecules (IL1β, IL-18, IL-23, EBI-3, IL-12, IL-35, TNF-α, and IL-6) or molecules involved in cell growth or immune responses (ICAM-1, MCP-1, VEGF) by ECs (80, 109, 110). On the contrary, production of anti-inflammatory molecules TGF-β, IL-10, and IL-37 is decreased (109). These downregulation or upregulations are reverted by several drugs including rosuvastatin, ezetimibe, rivaroxaban, and atorvastatin, all known to also targeting the cholesterol metabolism (109, 110, 111). As mentioned previously, 25-HC increase provokes a loss of the lung EC barrier integrity by disorganizing VE-cadherin assembly (54). This toxicity on EC alters the endothelial barrier permeability by affecting junctions between cells (54, 109). It is however possible to restore the barrier integrity by using for example the glucose-lowering drug inhibiting sodium-glucose cotransporter 2 (108). This inhibitor is newer and has shown many cardiovascular benefits. 25-HC also enhances EC apoptosis and impairs endothelium-dependent vasodilation (106). 25-HC has been also shown to be produced by vascular smooth muscle cells in response to the vasoactive angiotensin II peptide hormone (112) and induces reactive oxygen species generation, mitochondrial activation and cell death (113, 114, 115). 25-HC also triggers vascular calcification by activating ER stress (116). With all these considerations in mind, this is evident that the involvement of 25-HC in cardiovascular diseases, atherosclerosis and more globally to vascular defects, should be further investigated.

At the intestine level, *CH25H* contributes to the development of intestinal fibrosis (117) and its expression is increased in experimental colitis mice (118) but also in inflammatory bowel disease patients (119). Inflammatory bowel disease encompasses Crohn’s disease and ulcerative colitis in which defects of the intestinal epithelial barrier are reported (120). *Ch25h*^−/−^ mice with induced colitis exhibited aggravated colitis scores and, lower tight junction protein expression as well as higher levels of IL-6 (118). Addition of exogenous 25-HC to the mice diet alleviated disease symptoms and reversed the above-mentioned observations. Experiments done in vitro with human intestinal cells also reported that high concentrations of 25-HC induce tight junction genes expression (118).

Altogether these data indicate that 25-HC and probably its metabolite can act at the level of ECs and EPs in response to inflammatory situations, thus modifying endothelial/epithelial barrier properties. 25-HC decreases or increases permeability, in epithelial and endothelial barrier, respectively, that it suggests that this oxysterol can have an organ-dependent role and different molecular pathways. Because SARS-CoV-2 also infects intestinal cells (121), it is likely that 25-HC also plays an important role in gut protection against viral infection. SARS-CoV-2 also infects ECs and alters endothelial permeability (122, 123), but the effect of 25-HC in this process has never been investigated to our knowledge.

## 25-HC in cancers

Oxysterols likely play a central role in cancer onset and development but its remains to be clarified. Indeed, since decades, several studies have reported an increased oxidation of cholesterol, and therefore an increased 25-HC production, in different subsets of cancer cells (124). However, more recent investigations reported protective effects of 25-HC in tumor growth and metastasis onset. For example, in pancreatic cancer cells, methylation of the *CH25H* gene decreases its expression and is associated with poor prognosis. Despite the fact that there is no data on 25-HC levels, it is assumed that low levels of 25-HC promote tumor growth in this organ (125).

As described above, 25-HC shows powerful inhibition activity against viral infection. Human papilloma viruses are involved in cervical cancers onset and development, thus threatening women's health. 25-HC treatment of cervical epithelial-derived HeLa and C-33A cells provokes cytoskeleton remodeling, thus decreasing cells infection (126). This actin remodeling is mediated by activation of the Rho/ROCK/LIMK/cofilin axis and leads to cell death in colorectal cancer cell spheroids (127).

Tumor-derived extracellular vesicles (TEVs) have been shown to promote metastasis formation in melanoma, in particular. TEV from patients suppress interferon-mediated responses, and therefore *CH25H* expression. This decrease of CH25H expression is correlated with metastasis and poor survival in patients (128), so 25-HC seems to have protective effects. In turn, it has been shown that 25-HC suppresses TEV formation and uptake by targeted cells, including ECs (129). These data indicate that 25-HC displays some anti-metastatic properties through the suppression of angiogenesis processes.

25-HC has also been found to be involved in the progression of breast and ovarian tumors by activating the estrogen receptor α–mediated signaling pathway (130) and by promoting resistance to antihormone treatment in ER-positive breast cancer (131). However, investigation of 25-HC levels in 2,282 women with breast cancer diagnosis associated higher levels of 25-HC with lower risk of recurrence in patients, suggesting that the role of 25-HC in breast cancer remains to be clarified (132).

On the other side, 25-HC has been reported to promote the migration and invasion of adenocarcinoma cells in lung (133) as well as gastric cancer cell migration and invasion ability by upregulating TLR2-NF-κB–mediated matrix metalloproteinases expression (134). Therefore, we can speculate that exact role of 25-HC in cancer onset and development might be dependent on the targeted organ, as well as the origin of the tumorigenesis process, in particular if viral infection is involved.

## 25-HC in the CNS

Besides the roles of 25-HC and 7α,25-DHC in immune response through the immune cells migration, it has been hypothesized that these oxysterols might also have pivotal roles in central nervous disorders, particularly in multiple sclerosis (MS) characterized by demyelination processes and immune cells transmigration in CNS. 25-HC levels increased in plasma of MS patients in a 5-year follow-up study (135) but is decreased in relapsing-remitting MS patients compared to controls (136). Using a mouse model of MS, the experimental autoimmune encephalomyelitis (EAE) model, it has been demonstrated that levels of Ch25h, 25-HC, and 7α,25-DHC are increased in CNS (57, 66, 137), including at the level of the ECs composing BBB (58). In fact, 25-HC and 7α,25-DHC might control or participate together in the responses to neuroinflammatory processes in MS but their roles remain debated. In *Ch25h*-deficient mice EAE disease course is attenuated when compared with WT littermates (66), whereas other studies reported on the contrary that *Ch25h*-KO mice display an exacerbated EAE when compared to heterozygote control mice (138). 25-HC and 7α,25-DHC drive proinflammatory lymphocytes (EBI2 Th17–expressing cells) trafficking (66), probably across the BBB. The sole deletion of *Ch25h* expression in BBB EC dampens EAE (58), and favors polymorphonuclear myeloid-derived suppressive cells expansion and infiltration across the BBB. These cells have been described before mainly in the context of cancer (58).

Elevated concentrations of 25-HC are also measured in cerebrospinal fluid of patients with inflammatory CNS disease classified as suspected autoimmune disease (136).

Human and mouse astrocytes express EBI2 and the enzymes necessary for synthesis and degradation of 7α,25-DHC (59) and treatment of these cells with 7α,25-DHC triggers cell migration (59). Additionally, expression of EBI2 is increased in astrocytes and microglial cells in MS lesions (139), as well as in oligodendrocytes during their maturation (140). In this latter cell type, LXR activation by 25-HC promotes myelin gene production, myelination as well as oligodendroglial cell maturation (40). In rats, levels of 25-HC increase after spinal cord injury and contribute to migration of microglia/macrophages to the injured site (141). EBI2 activation by 7α,25-DHC attenuates neuronal demyelination and inhibits release of proinflammatory cytokines (142). Altogether, these data highlight positive roles for 25-HC, EBI2 and 7α-25DHC in myelin formation and suggest that they might have protective functions under pathophysiological conditions.

Cholesterol transport within the CNS as well as lipoprotein genesis are two processes deeply altered in several neurodegenerative diseases including AD. The major part of the studies focusing on the role of oxysterols in AD were focused on 24S-HC. However, few studies suggest that role of 25-HC in brain CNS homeostasis and AD would deserve further investigations. For example, when microglial cells are stimulated and activated by IL-1β, they produce 25-HC that in turn act at the astrocyte level by paracrine action to promote cholesterol efflux to ApoE particles (65, 143). Furthermore, *Ch25h* was found to be overexpressed in brains of AD patients as well as in mouse models of amyloid deposition and tau-mediated neurodegeneration (65) and 25-HC levels are increased in cerebrospinal fluid of late-stage AD patients, as well as in AD brains tissue and mitochondria (144). Treatments with 25-HC increased internalization of amyloid β peptides (Aβ peptides) by neural cells and its accumulation to the ER (145), probably because this oxysterol increases association of these peptides with the membranes (146).

At the direct neuronal level, recent studies have investigated the effects of *Cyp7b1* deletion in mice and reported few or no impact on cognitive functions and neuronal morphology despite the high level of 25-HC in plasma (147, 148). However, direct treatment of neurons with 25-HC decreases neurites, neurons viability, metabolism, and disrupts hippocampal synaptic transmission via N-methyl-D-aspartate **receptor**-mediated metaplasticity (149, 150). In a mouse model of stroke, 25-HC alleviated the lesions by inhibiting autophagy as well as by reducing brain nerve cell apoptosis (151).

Therefore, several lines of evidence indicate that 25-HC and 7α,25-DHC are implicated in CNS homeostasis and neurological disorders. A number of questions remain unanswered. This is not clear for example, if peripheral 25-HC can reach the CNS though the BBB or if this is in situ CNS production that act in brain functioning. In addition, if 25-HC and its metabolite act in noninflammatory states is also crucial to be determined.

## 25-HC in macular degeneration

Age-related macular degeneration (ARMD) is the leading cause of blindness in the elderly population in developed countries. As for AD and atherosclerosis, ARMD is closely associated with altered lipid metabolism, oxidative stress, and therefore oxysterols. Aβ peptides deposition is also a key event in ARMD and studies performed in retinal pigment EPs demonstrated that these peptides promote 25-HC and 27-HC formation via oxidative stress (152). In turn, oxysterols produce Aβ production that suggests a cytotoxic cascade that inevitably leads to ARMD (153). Aβ peptides and 25-HC conjointly induce oxidative stress and cell death, involving an activation of P2X7-pannexin-1 receptors that form large nonselective pores at the cell membranes, thus resulting in inflammation through the inflammasome, oxidative stress, and, ultimately, cell death especially by apoptosis (152). Chromatin condensation is also observed after 25-HC stimulation but this latter process requires a P2X7 receptor-pannexin-1–independent pathway (152). In addition, 25-HC induces inflammatory events via IL-8 secretion dependent of activation of the ME/ERK signaling pathway (154). 25-HC also promotes VEGF production and secretion that leads to the proliferation of ECs as previously mentioned (154).

## 25-HC and 7α,25-DHC in osteoporosis and osteoarthritis

25-HC and 7α,25-DHC also contribute to osteoporosis and osteoarthritis, two other age-related disorders closely associated with altered cholesterol metabolism. Osteoarthritis is a degeneration of articular cartilage in synovial joints and osteoporosis is an asymptomatic bone disease that can increase the risk of bone fractures through the change in bone quality or structure caused by the decrease in bone mineral density and mass. One of the major factors responsible for osteoporosis is a deficiency in estrogen levels. This leads to inflammatory processes and oxidative stress that in turn inhibit osteoblast differentiation, proliferation, and promote apoptosis, as well as osteoclast activity. Surgical ovariectomy of female mice increases CH25H expression and cultivation of human osteoblast-like MG-63 cells in inflammatory conditions increases 25-HC production (155). When added in vitro, 25-HC triggers apoptosis of MG-63 cells and increases the autophagy process via a crosstalk between p53 and Akt cellular signaling pathways (155). This 25-HC–mediated cell death being closely linked to oxidative stress, and autophagy, is sometimes defined as oxiapoptophagy. Interestingly, this oxiapoptophagy is also promoted in fibroblasts after 25-HC and 7α,25-DHC treatment (156, 157). Furthermore, 25-HC and 27-HC promoted osteoclastogenesis by the upregulation of nuclear factor of activated T-cell cytoplasmic 1 and Sp1 in mouse bone marrow macrophages stimulated with the cytokine RANKL and monocyte macrophage colony-stimulating factor (158).

Besides, human and mouse osteoarthritic chondrocytes show an upregulation of CH25H and CYP7B1 expression and an increase in oxysterol production (159). Adenoviral overexpression of *Ch25h* or *Cyp7b1* in mouse joint tissues caused experimental osteoarthritis, whereas knockout or knockdown of these hydroxylases abrogated the pathogenesis of osteoarthritis (159). Chondrocytes treated with inflammatory molecules show an increase of CH25H expression and 25-HC production (159, 160). Incubation of chondrocytes with 25-HC induces a caspase-dependent apoptosis (160).

Altogether, these data suggest 25-HC and 7α25-DHC may act as metabolic pathophysiological factors in osteoarthritis and osteoporosis that are closely linked to significant apoptosis and autophagy processes.

## Conclusion

Among all the existing oxysterols, 25-HC shows a unique synthesis process significantly regulated by inflammation, and an exclusive function consisting of reducing HMGCR and LDLR levels via inhibiting the SCAP/SREBP-2 pathway, and of activating the LXR signaling pathway. These exceptional properties make 25-HC as a key player of host defense during viral and bacterial infection. Moreover, 25-HC and its metabolite 7α,25-DHC have also shown interesting properties by modulating immune cell migration or endothelial/epithelial barrier properties, thus involving them in atherosclerosis and CNS disorders. 25-HC also shows great importance in age-related diseases often associated with altered cholesterol metabolism, oxidative stress, inflammation, and cell death like AD, atherosclerosis, osteoporosis, or macular degeneration (154, 161, 162). 25-HC is therefore a very promising oxysterol with huge therapeutic potential. Indeed, it might be beneficial to target 25-HC in order to prevent or to dampen the onset and development of these diseases. However, beneficial and deleterious effects are reported for 25-HC in animal and cell models. It is, therefore, not clear if 25-HC levels need to be increased or decreased to treat a disease, and whether inflammatory states are necessary.

Because many cell-types in humans express the enzymes involved in 25-HC synthesis and degradation, it is highly likely that undiscovered functions for 25-HC will be highlighted in the near future. For example, it has been recently demonstrated that 25-HC might have beneficial effects on hepatic steatosis (73) and that 25-HC levels are increased in nonalcoholic fatty liver disease patients, thus suggesting that it can serves as indicators of nonalcoholic fatty liver disease onset and progression (163). Its potential role in this disease remains to be investigated, but the dosage of 25-HC as biomarker was also recently raised by another study quantifying lipid profile in plasma and urine of adjuvant-induced arthritis rats in which urine levels of 25-HC were decreased in diseased animals versus the control (164).

In addition, recent studies have also showed that 25-HC, but also cholesterol, binds to the extracellular domain cysteine rich and activates the SMOOTHENED protein that plays a pivotal role in the hedgehog signaling pathway (165, 166, 167). This signaling pathway is essential during embryonic development and controls proper tissue function and repair in adults. Defects in hedgehog pathway leads to congenital abnormalities or dead in early stages, but also cancers in late stages. It remains therefore compulsory to deeply investigate the role of 25-HC and 7α,25-DHC in development and maintenance signaling pathways in humans.

Therefore, future studies on 25-HC metabolism and effects represent exciting perspectives to better understand some diseases and to develop new therapeutic approaches or biomarker tools.

## Conflict of interest

The authors declare that they have no conflicts of interest with the contents of this article.
